# Biomechanical stability of oblique lateral interbody fusion combined with four types of internal fixations: finite element analysis

**DOI:** 10.3389/fbioe.2023.1260693

**Published:** 2023-09-25

**Authors:** Jiayu Hao, XianSheng Tang, Nizhou Jiang, Hong Wang, Jian Jiang

**Affiliations:** ^1^ Department of Spine Surgery, Dalian Municipal Central Hospital, Dalian University of Technology, Dalian, China; ^2^ Department of Engineering Mechanics, Dalian University of Technology, Dalian, China

**Keywords:** finite element analysis, OLIF, internal fixation, biosolid mechanics, CAGE

## Abstract

**Objective:** Using finite element analysis to identify the optimal internal fixation method for oblique lateral lumbar interbody fusion (OLIF), providing guidance for clinical practice.

**Methods:** A finite element model of the L4 – L5 segment was created. Five types of internal fixations were simulated in the generated L4-L5 finite element (FE) model. Then, six loading scenarios, i.e., flexion, extension, left-leaning, right-leaning, rotate left, and rotate right, were simulated in the FE models with different types of fixations. The biomechanical stability of the spinal segment after different fixations was investigated.

**Results:** Regarding the range of motion (ROM) of the fused segment, OLIF + Bilateral Pedicle Screws (BPS) has a maximum ROM of 1.82° during backward bending and the smallest ROM in all directions of motion compared with other models. In terms of the von Mises stress distribution on the cage, the average stress on every motion direction of OLIF + BPS is about 17.08MPa, and of OLIF + Unilateral Vertebral Screw - Pedicle Screw (UVS-PS) is about 19.29 MPa. As for the von Mises stress distribution on the internal fixation, OLIF + BPS has the maximum internal fixator stress in left rotation (31.85 MPa) and OLIF + Unilateral Pedicle Screw (UPS) has the maximum internal fixator stress in posterior extension (76.59 MPa). The data of these two models were smaller than those of other models.

**Conclusion:** OLIF + BPS provides the greatest biomechanical stability, OLIF + UPS has adequate biomechanical stability, OLIF + UVS-PS is inferior to OLIF + UPS synthetically, and OLIF + Double row vertical screw (DRVS) and Individual OLIF (IO) do not present significant obvious advantages.

## Introduction

Currently, lumbar fusion surgery (LIF), including posterior lumbar fusion (PLIF) or transforaminal lumbar fusion (TLIF), is widely used in clinical practice (Huang et al., 2022). However, insertion of the fusion through a posterior approach requires the removal of the posterior structures of the vertebral body, which can affect vertebral stability. Additionally, repeated traction on the dural sac and nerve roots during the procedure may lead to nerve injury. Multiple surgical approaches have been attempted to minimize complications.

Oblique lateral lumbar interbody fusion (OLIF) is considered one of the best options for lumbar fusion (Aleinik et al., 2021). In 1997, Mayer (Mayer, 1997) described a minimally invasive anterior approach to the lumbar spine through a retroperitoneal approach at the L2-L5 level and a transabdominal approach at the L5-S1 level, which first proposed OLIF. In OLIF, the cage enters the disc from the oblique lateral side, preserving the small posterior joints, muscles, and other tissues. In 2012, Silvestre et al. (Silvestre et al., 2012) improved Mayer’s approach to the procedure, resulting in the OLIF that is currently used. OLIF is a discectomy implant fusion performed through the anatomical gap between the retroperitoneal lumbar major muscle anteriorly and the aorta. The mechanisms of OLIF include restoring disc height, increasing posterior longitudinal ligament tension, and improving the sagittal sequence of the spine. (Alimi et al., 2014; Liu et al., 2019).

Compared to other lumbar fusion methods, OLIF has the following advantages (Huang et al., 2022; Liu et al., 2022; Zhang et al., 2022). First, OLIF allows the cage to enter the lumbar spine anteriorly without opening the spinal canal or damaging the posterior muscles, ligaments, and bony structures. This preserves more bone, which is particularly important for patients with disc degeneration combined with osteoporosis. Second, by removing sufficient disc tissue and providing a large contact area with the endplate, the fusion device significantly enhances the supporting strength of the fusion. Third, OLIF reduces the possibility of damaging the lumbar muscles and lumbosacral nerves. Fourth, the interbody fusion used in OLIF is much larger compared to conventional posterior fusions, and it is placed across the endplate, which significantly enhances its biomechanical stability. In addition, OLIF has the advantages of a low complication rate, less surgical blood loss, shorter operative time, and shorter patient hospital stay (Mobbs et al., 2015; Lu and Lu, 2019; Aleinik et al., 2021). Joseph et al. (2015) reported a complication rate of 20.2% (380/1885) after TLIF, whereas Abe et al. (2017) reported a complication rate of 1.2% (2/155) after OLIF, indicating a significant reduction in innerve injury with OLIF. Moreover, the OLIF procedure preserves more of the anatomy, theoretically providing more resistance during motion (Kim et al., 2005).

It has also been suggested that OLIF leads to lumbar instability and increases the risk of fusion subsidence and fracture (Abe et al., 2017; Quillo-Olvera et al., 2018; Bereczki et al., 2021). After OLIF, especially in osteoporotic patients, surgical injury can lead to instability of the corresponding lumbar segment, subsidence, and displacement of the fusion cage, which can ultimately result in surgical failure (Malham et al., 2015). Therefore, in most cases, OLIF requires reinforcement with internal fixation devices to enhance the stability of the fusion (Cappuccino et al., 2010; Shasti et al., 2019). The internal fixation system must maintain good function until firm bony fusion is achieved. For lumbar fusion, the stiffness of the internal fixation system at the operative segment and its ability to share the load of the fusion apparatus is fundamental to bone healing or fusion. The combination of fusion inserted through OLIF with posterior internal fixation instrumentation results in a stronger and more stable structure (Kim et al., 2005; Niemeyer et al., 2006; Kornblum et al., 2013). Different types of internal fixation devices play important roles in maintaining the stability of the operated segment and reducing fusion device complications (Pham et al., 2016; Xu et al., 2018). Fusion device complications are related to bone density, fusion level, disc position, disc height, and pedicle screw internal fixation (Kim et al., 2013; Oh et al., 2017). Fusion settling is a major factor in revision surgery after OLIF (Alimi et al., 2014; Tempel et al., 2018). Local healing is better facilitated if the load transmitted through the fusion device can be increased without fusion settling.

There is limited research on the biomechanical stability of OLIF combined with internal fixation. This study aims to identify an OLIF supplementary internal fixation method that can provide the best spinal stability. We established a normal vertebral body model and five surgical models and compared their biomechanical stability using finite element (FE) analysis. In these 5 models, although the Individual OLIF (IO) model did not have any additional internal fixation, we still established its model as a reference.

## Methods

### Three-dimensional FE model of the lumbar spine

A female volunteer (age: 39 years old, height: 169.0 cm, weight: 60.0 kg) with lumbar degenerative disease was recruited. The entire lumbar spine was scanned using a NEUVIZ 64-row spiral CT scanner with a slice thickness of 0.1 mm. Appropriate gray values were selected to distinguish bone and tissues. The images of the L4-L5 segment were selected from the complete lumbar spine image, as this is the most commonly used in OLIF surgery. The computed tomography images were stored in the format of Digital Imaging and Communications in Medicine (DICOM). The DICOM data were imported into Mimics Research 20.0 (Materialise, Belgium) for the three-dimensional (3D) reconstruction of Lumbar 4-5 segments. Then the reconstructed model was imported into Geomagic Wrap 2021 (Reverse Engineering Software, United States) for surface optimization, eliminating defects in the initial model. After the smoothing process was completed, the vertebral body of the spine model was offset inward by 0.5 mm. The hollow part between the original model and the offset model was added to the anterior vertebral body as cortical bone and the inner body was used as cancellous bone. The cortical bone on the upper and lower surfaces of the vertebral body is set as an endplate with a thickness of 0.5 mm. The cartilage part was first created by creating an appropriately sized cylinder in SolidWorks 2019 (CAD software, Dassault Systems, United States), and then performing Boolean operations on the L4 and L5 endplates that fit with the cylinder to generate the cartilage. The fusion device and bone screw were established in Solidworks 2019 with corresponding dimensions. Finally, all the models were imported into 3-material (Metric, Belgium) to adjust the screw positions, perform Boolean operations, generate four screw layout schemes, and import the inp file into Abaqus 6.14 after generating the mesh. The ligaments were established in Abaqus 6.14 with seven major ligaments created at appropriate locations: Anterior Longitudinal Ligament (ALL), Posterior Longitudinal Ligament (PLL), Ligamentum Flavum (LF), Intertransverse Ligament (ITL), Supraspinous Ligament (SL), and Interspinous Ligament (ISL).

In the interaction setting, the bonding surface of cortical bone and cancellous bone were bound, the fusion device was bound to the lower surface of the L4 segment and the upper surface of the L5 segment, the ligament was bound to the outer surface of cortical bone, the bone screw was bound to the surface of the hole after Boolean operation of the spine. The friction contact coefficient between the cartilage and the surface of the upper and lower articular processes was set to 0.1 (Cai et al., 2022). The bone parts in the complete FE model of the L4-L5 segments include cortical bone, cancellous bone, and cartilage parts. The cortical bone thickness was set to 0.5 mm based on CT image estimation and another research (Cai et al., 2022). The element type for cortical bone, cancellous bone, fusion cage, and bone screw was C3D4, the element type of ligament was T3D2, and the element type of cartilage was C3D4. The developed L4-L5 spinal segment model was meshed using C3D4 elements after a mesh convergence study.

### FE models of the internal fixation and cage

**FIGURE 1:**
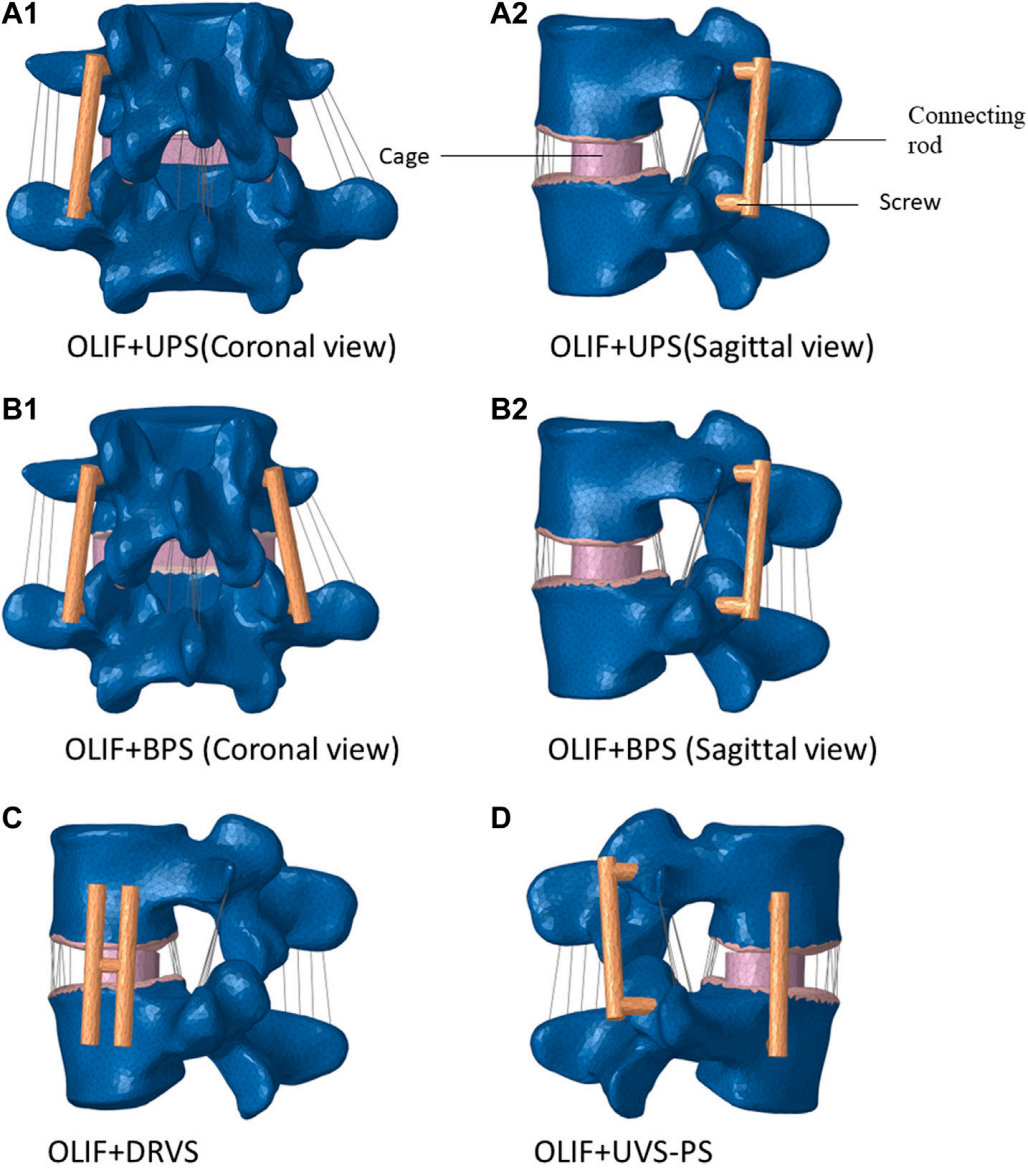
Four types of internal fixation models. (OLIF: Oblique lateral lumbar interbody fusion, UPS: Unilateral Pedicle Screw, BPS: Bilateral Pedicle Screws, DRVS: Double row vertical screw, UVS-PS: Unilateral Vertebral Screw-Pedicle Screw).

In the present study, the Individual OLIF (IO) model and OLIF combined with four internal fixation models were established (Figure 1). The cage measures 45.0 mm in length, 17.0 mm in width, and 14.0 mm in height. The length of the screw is 45.0 mm and the diameter is 6.5 mm. The connecting rod has a length of 50.0 mm and a diameter of 5.5 mm. The fusion device and internal fixation were established by corresponding dimensions in Solidworks 2019. During the FE simulation, the entire nucleus pulposus and fibrous ring were removed. In the Individual OLIF (IO) model, fusion cages were implanted in the intervertebral space, and no internal fixation was implanted. In the OLIF + Unilateral Pedicle Screw (OLIF + UPS) model, while implanting a fusion cage in the intervertebral space, screws were implanted in one side of the pedicle, and the upper and lower screws were connected by connecting rods. In the OLIF + Double row vertical screw (OLIF + DRVS) model, while implanting a fusion cage in the intervertebral space, double row screws were implanted in the lateral vertebral body, which was connected by connecting rods. At the same time, a transverse connection was added at the level of the intervertebral disc to connect the bilateral connecting rods. In the OLIF + Unilateral Vertebral Screw - Pedicle Screw (OLIF + UVS-PS) model, a fusion cage was implanted in the intervertebral space, screws were implanted in one side of the pedicle, and screws were implanted in the same side of the vertebral body. The pedicle screws and vertebral screws were connected by connecting rods, respectively. In the OLIF + Bilateral Pedicle Screws (OLIF + BPS) model, a fusion cage was implanted in the intervertebral space, screws were implanted in both pedicle screws, and the upper and lower screws were connected by connecting rods.

### Definition of the properties of the materials

Linear elastic material models were used for the bony tissues. Facet cartilage and intervertebral discs were modeled using Neo-Hookean and Mooney-Rivlin hyperelastic materials (Du et al., 2014; Cai et al., 2020; Cai et al., 2022; Liu et al., 2022). The nucleus pulposus constituted 50% of the disc and the cortical bone thickness was 0.5 mm (Cai et al., 2022; Zhang et al., 2022). The material parameters of the tissue model are shown in Table 1. The material used in the cage is Poly ether ether ketone (PEEK), and the material used for internal fixation is titanium alloy. Linear elastic material models were used for the spinal cage and internal fixation components. Therefore, an elastic modulus of 22000 MPa and Poisson’s ratio of 0.3 were defined for the cage, and an elastic modulus of 110000 MPa and Poisson’s ratio of 0.3 were defined for internal fixation. All ligaments were meshed using tension truss elements (T3D2). Based on literature data, the bilinear elastic material model described the mechanical behavior of ALL, PLL, LF, ITL, SSL, and ISL, while the linear elastic material model described the mechanical behavior of CL. (Table 2).

**TABLE 1:** Material properties, element type, and number for the component in the FE model of the spinal segment.

Component	E [MPa]	ν	Element type	References
Bony Structures				
Cortical bone	12,000	0.3	C3D4	Burstein et al. (1976)
Cancellous bone	1,500	0.3	C3D4	Lindahl (1976)
Posterior bone	3,500	0.3	C3D4	Shirazi-Adl et al. (1986)
End plate	12,000	0.3	C3D4	Grant et al. (2001)
Facet cartilage	20	0.3	C3D4	
Intervertebral disc				
Annulus fibrosus	Calibrated stress-strain curves			
Nucleus pulposus	Mooney–Rivlin, C1 = 0.12, C2 = 0.03			
Implants				
Cage	22,000	0.3	C3D4	
Internal fixation	110,000	0.3	C3D4	

(*E represents Young’s modulus and ν represents the Poisson’s ratio).

**TABLE 2:** Material properties, element type, number, and cross-sectional area of the ligaments in the FE model.

Ligament	E1*[Mpa]	E2*[Mpa]		ε12**	Element type (number)	Area [mm^2^]	Reference
Anterior Longitudinal Ligament	7.8	20.0		0.12	T3D2 (7)	32.4	Moramarco et al. (2010)
Posterior Longitudinal Ligament	1.0	2.0		0.11	T3D2 (6)	5.2	
Ligamentum Flavum	1.5	1.5		0.06	T3D2 (3)	84.2	
Intertransverse Ligament	10.0	59.0		0.18	T3D2 (3)	1.8	Chazal et al. (1985)
Supraspinous Ligament	3.0	5.0		0.2	T3D2 (4)	25.2	
Interspinous Ligament	13.2	42.6		0.15	T3D2 (4)	35.1	
Ligament	E [Mpa]	ν		Element type (number)		Area [mm2]	Reference
Capsular Ligament	24.4	0.3		T3D2 (6)		23.8	Tyndyka et al. (2007)

(*E1 denotes the Young’s modulus of the first phase and E2 denotes the Young’s modulus of the second phase; ε12** denotes the strain transition between two bilinear moduli E_1_ and E_2_.).

### The setting of the loading and boundary conditions

**FIGURE 2:**
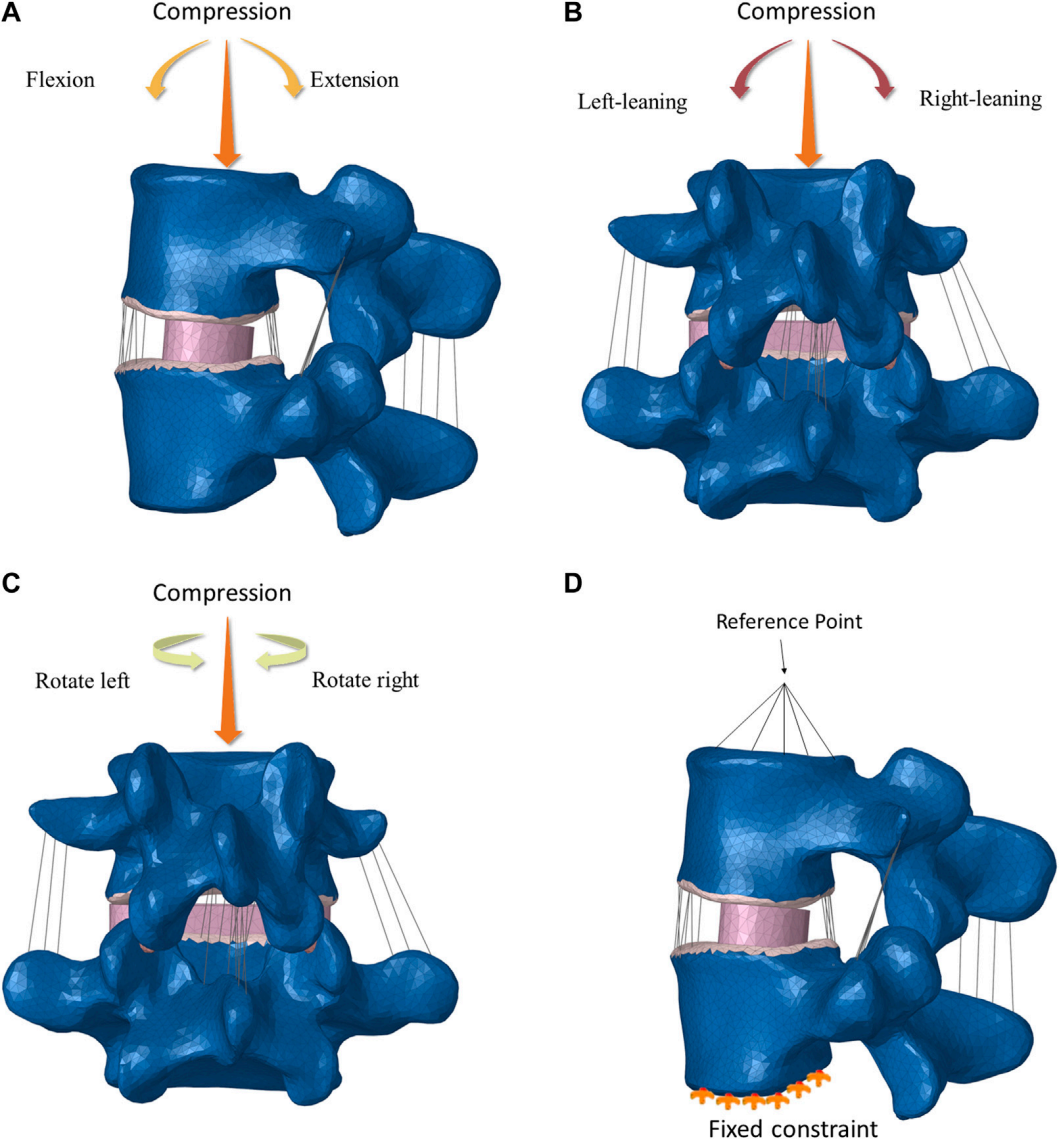
Six activity directions and boundary conditions.

In the FE L4-L5 spinal fixation models, the spinal cage was fully constrained to its adjacent bony parts. The screws were fully constrained to their surrounding bone tissues, and the connecting bars and screws were also fully constrained to each other. Six loading scenarios (flexion and extension, leaning-left and leaning-right, rotate right and rotate left) were simulated in the FE L4-L5 spinal fixation models to mimic the daily activities (Figure 2). In all the loading scenarios, a vertically downward load of 200.0 N was applied in the model to simulate the body weight. Boundary conditions were established to fix the lower surface of L5, a reference point was established at the center of the upper surface of L4, the reference point was coupled with the upper surface, a torque of 5 N m and a concentrated force perpendicular to the upper surface of the vertebral body of 200 N were applied to the reference point, and the forces on the spine were jointly simulated. After the settings were applied, the results were calculated.

IO model has 232,411 nodes and 1424600 elements. OLIF + UPS model has 228,770 nodes and 1393400 elements. OLIF + DRVS model has 232,840 nodes and 1415121 elements. OLIF + UVS-PS model has 229,094 nodes and 1390833 elements. OLIF + BPS model has 242,659 nodes and 1808364 elements. The mesh size of bones ranges from 0.5 to 2.5. The mesh size of the cage ranges from 1.2 to 4.5. The mesh size of the screw ranges from 0.5 to 2.5.

## Results

### Validation of the FE spinal model

**FIGURE 3:**
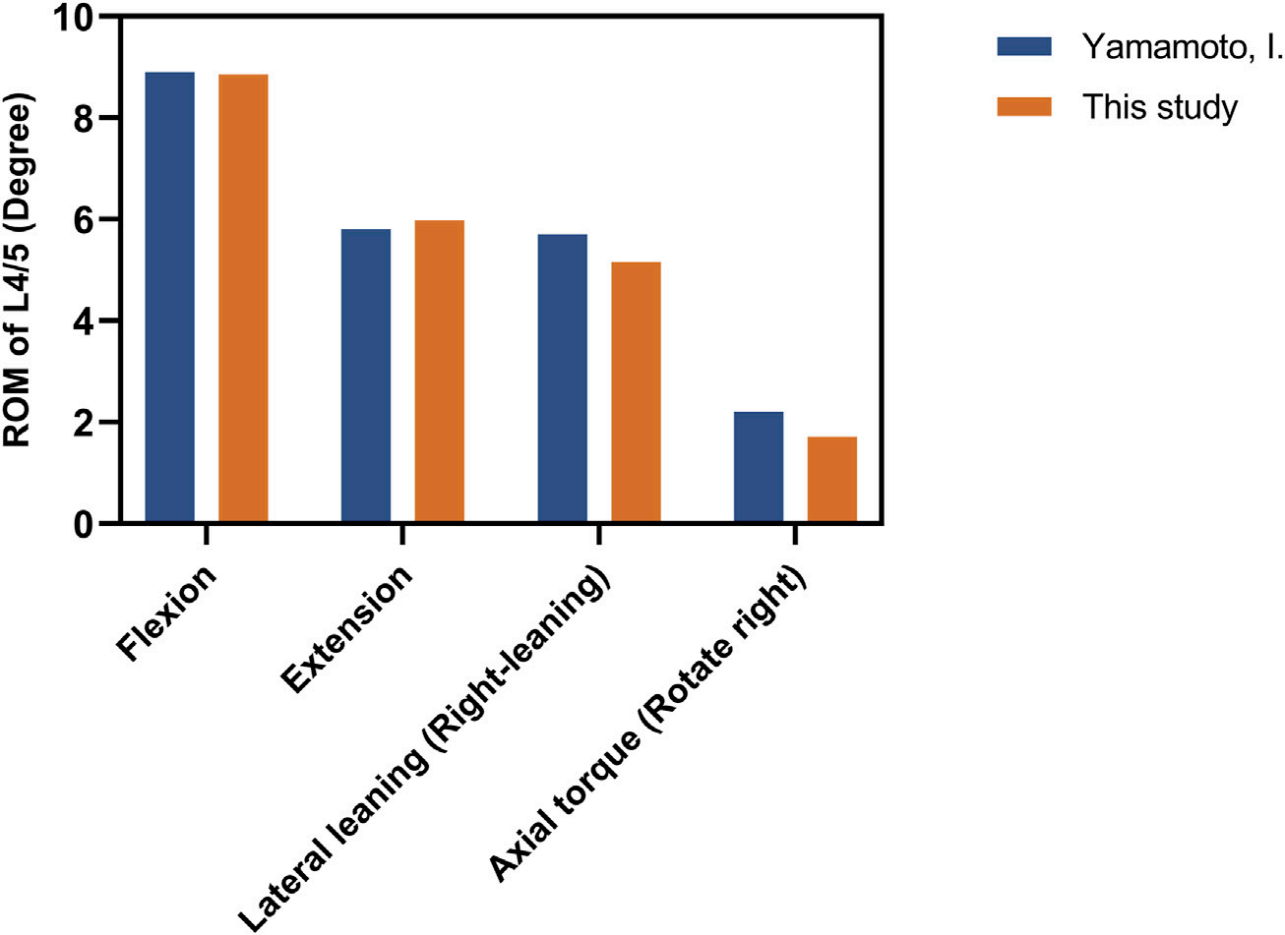
The comparision of ROM between the intact model and the previous *in vitro* experimental study. (Unit:degree).

Apply 10 N m to our model and compare the ROM of fused segments with cadaver studies (Yamamoto et al., 1989). Our results fall within the range of variation, demonstrating our model’s reliability (Figure 3).

### Ranges of motion (ROM) of the fused spinal segment

**FIGURE 4:**
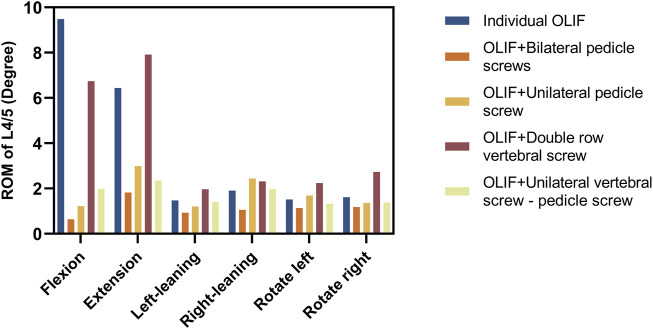
The ranges of motion (ROM) of fusion segments (Unit:degree).

The ultimate goal of OLIF surgery is to make the fusion segment as a whole part, and thus the angle of vertebral body movement can directly reflect the effectiveness of the surgery. The ROM of the fused segment is expressed as an angle, which directly reflects the stability of the fused segment. The less ROM, the more stable of fusion segment and the less risk of complication (Cai et al., 2022). The ROM of the surgical model under a combined load of 200 N and 5 N m is shown in Figure 4 and Table 3. In the flexion group, the ROM of IO was 9.48°, and the ROM of OLIF + DRVS was 6.74°. In the backward bending group, the ROM of IO was 6.44°, and the ROM of OLIF + DRVS was 7.91°. These four data are significantly greater than the data of other models in various motion directions. OLIF + BPS has a maximum ROM of 1.82° during backward bending and has the smallest ROM in all directions of motion. OLIF + UPS is similar to OLIF + UVS-PS, and both models are slightly inferior to OLIF + BPS overall, but the difference is not very significant.

**TABLE 3:** The ROM of fusion segments (Unit: degree).

Name	Flexion	Extension	Left-leaning	Right-leaning	Rotate left	Rotate right
Individual OLIF	9.48	6.44	1.47	1.90	1.51	1.61
OLIF + Bilateral pedicle screws	0.64	1.82	0.93	1.05	1.14	1.18
OLIF + Unilateral pedicle screw	1.22	2.99	1.20	2.44	1.69	1.36
OLIF + Double row vertebral screw	6.74	7.91	1.96	2.31	2.24	2.73
OLIF + Unilateral vertebral screw - pedicle screw	1.98	2.35	1.40	1.97	1.33	1.38

### Distribution of the von mises stress in the cage and internal fixations

**FIGURE 5:**
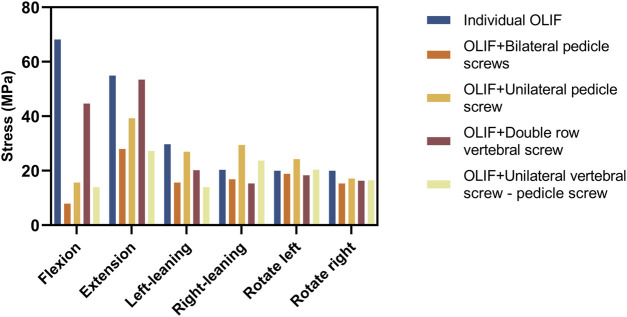
Maximum von Mises stresses distributed on the cage (Unit: MPa).

**FIGURE 6:**
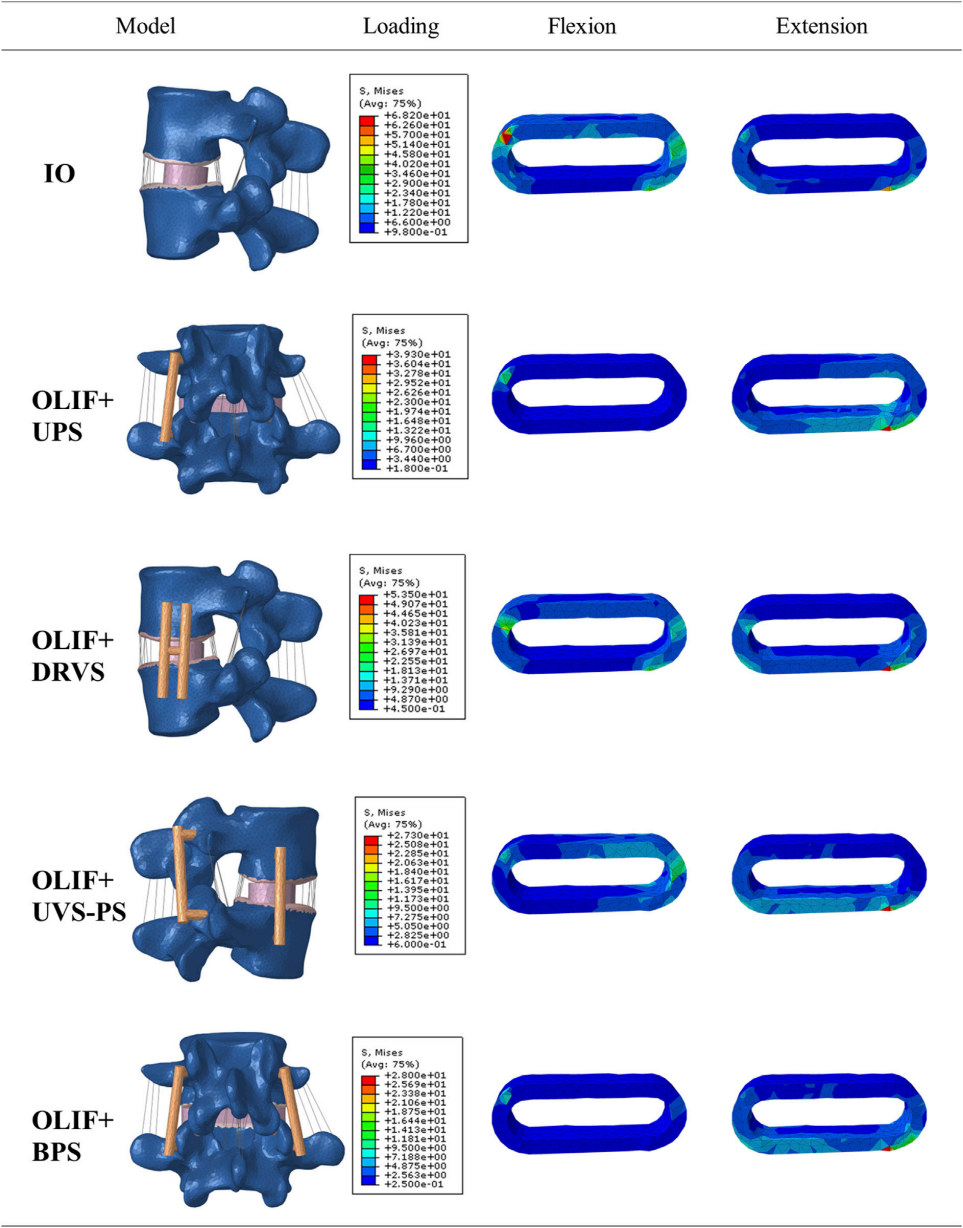
Cloud map of stress distribution in the fusion device during flexion and extension.

The greater the stress distributed on the fusion cage and internal fixation, the higher the likelihood of complications is to occur, such as cage settlement, endplate collapse, fractures, and screw loosening and fracture (Villa et al., 2014; Singhatanadgige et al., 2021; Qin et al., 2022). The distribution of the von Mises stress on the Cage is shown in Figures 5, 6; Table 4. In the flexion group, the von Mises stress of IO was 68.15 MPa, and of OLIF + DRVS was 44.64 MPa. In the backward bending group, the von Mises stress of IO was 54.07 MPa, and of OLIF + DRVS was 53.42 MPa. These four data are significantly greater than the data of other models in various motion directions. The data of OLIF + BPS and OLIF + UVS-PS are similar and are less than others. The average stress on every motion direction of OLIF + BPS is about 17.08 MPa and of OLIF + UVS-PS is about 19.29 MPa.

**TABLE 4:** The maximum stress of the cage (Unit: MPa).

Name	Flexion	Extension	Left-leaning	Right-leaning	Rotate left	Rotate right
Individual OLIF	68.15	54.87	29.72	20.29	19.98	19.99
OLIF + Bilateral pedicle screws	7.919	27.98	15.65	16.85	18.82	15.29
OLIF + Unilateral pedicle screw	15.63	39.23	26.96	29.42	24.24	17.11
OLIF + Double row vertebral screw	44.64	53.42	20.16	15.33	18.27	16.31
OLIF + Unilateral vertebral screw - pedicle screw	13.99	27.22	13.96	23.7	20.37	16.52

**FIGURE 7:**
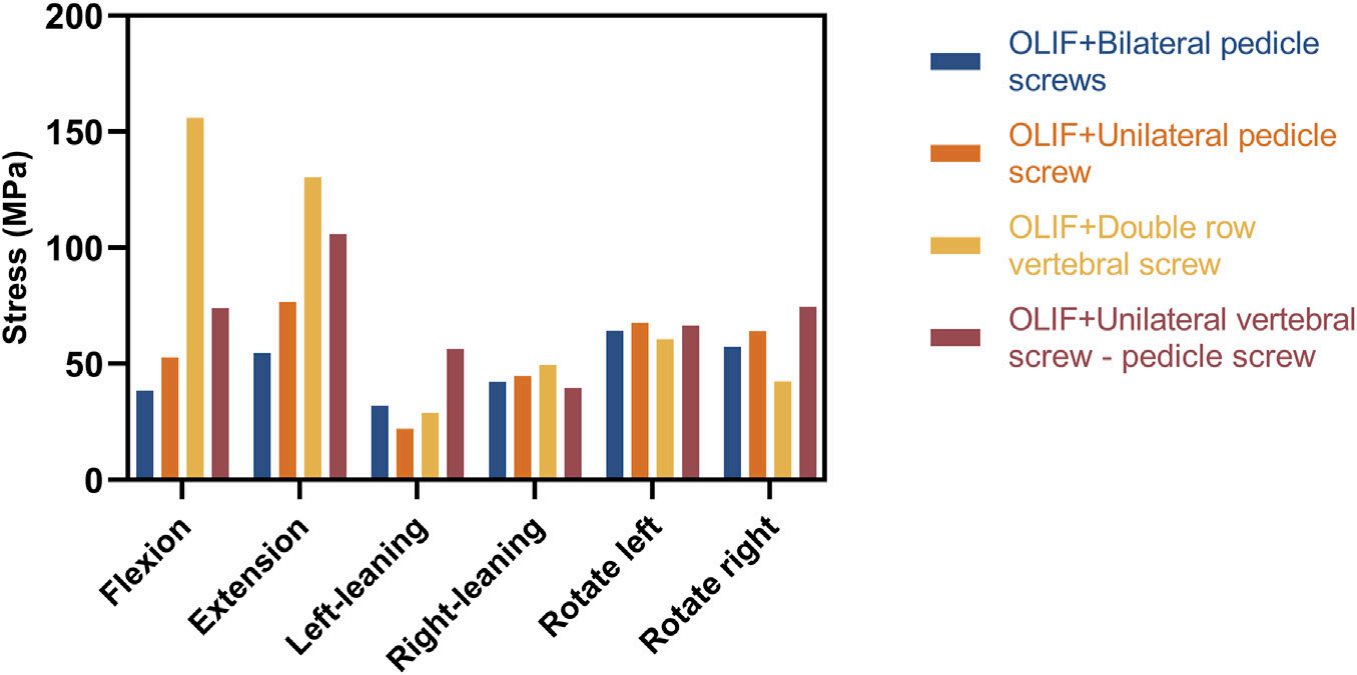
Maximum von Mises stresses distributed on the internal fixation sytem (Unit: MPa).

**FIGURE 8:**
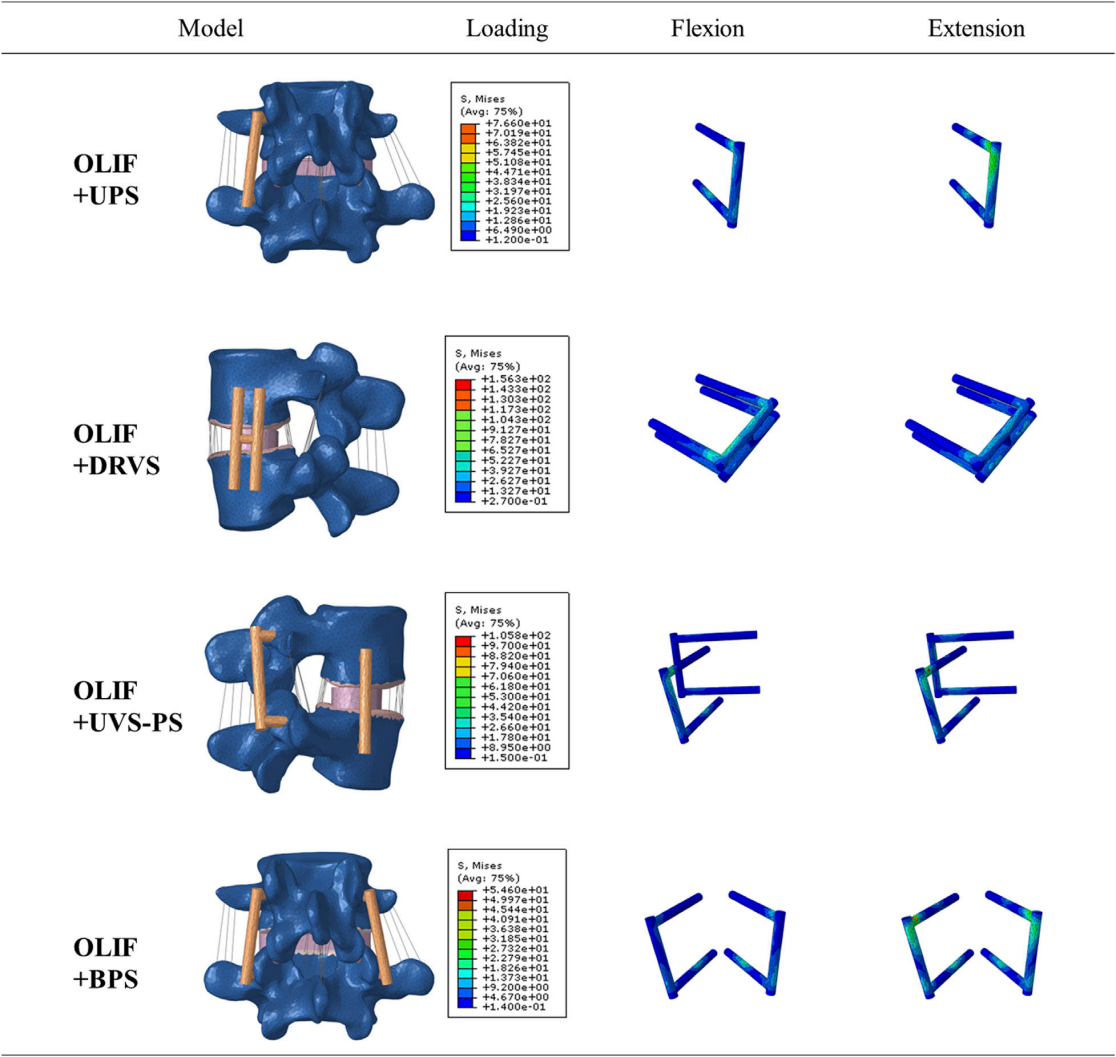
Internal fixation strss distribution cloud map during flexion and extension.

The distribution of the von Mises stress on the internal fixation is shown in Figures 7, 8; Table 5. In flexion and posterior extension, the maximum internal fixation stresses on OLIF + DRVS and OLIF + UVS-PS were significantly greater than those in the other models. OLIF + DRVS had the maximum internal fixator stress in forward bending (155.9 MPa), and OLIF + UVS-PS had the maximum internal fixator stress in posterior backward bending (105.8 MPa). OLIF + BPS had the maximum internal fixator stress in left rotation (31.85 MPa), and OLIF + UPS had the maximum internal fixator stress in posterior extension (76.59 MPa). The data of these two models were smaller than in the other models.

**TABLE 5:** Maximum stress on the internal fixation system (Unit: MPa).

Name	Flexion	Extension	Left-leaning	Right-leaning	Rotate left	Rotate right
OLIF + Bilateral pedicle screws	38.49	54.56	31.85	42.24	64.2	57.26
OLIF + Unilateral pedicle screw	52.82	76.59	21.98	44.78	67.52	64.13
OLIF + Double row vertebral screw	155.9	130.2	28.82	49.74	60.66	42.49
OLIF + Unilateral vertebral screw - pedicle screw	73.88	105.8	56.38	39.61	66.37	74.58

## Discussion

This study aims to identify an OLIF supplementary internal fixation method that can provide the best spinal stability. In the present study, the biomechanical stability of the L4-L5 spinal segment using different fixations was investigated using FE analysis. Five different models, e.g., OLIF + DRVS, OLIF + UPS, IO, OLIF + BPS, OLIF + UVS-PS, were simulated and the ROM of the fused spinal segment and stress distribution in the cage and internal fixations were investigated (Cai et al., 2022). All intervertebral discs in the figures have been replaced by fusion cages.

By comparing the ROM of different models, we found that OLIF + BPS has the least ROM in each motion mode. On the one hand, this indicates that OLIF + BPS provides a more stable load-sharing on the fusion, facilitates bone healing or fusion, and has the most stable fusion-vertebral body interface, reducing the likelihood of complications such as fusion loosening. On the other hand, the IO and OLIF + DRVS had the greatest ROM in flexion and extension motion compared to all surgical models. This implies that postoperative patients who accept one of these two operations are more likely to experience pain due to lumbar instability. The excessive motion indicates a weaker fusion-vertebral body interface, increasing the risk of cage loosening, displacement, or even prolapse.

The maximum stress on the IO and OLIF + DRVS fusion cages during flexion and extension is significantly greater than that on OLIF + BPS and OLIF + UVS-PS. This indicates that IO and OLIF + DRVS are more prone to fusion cage settlement compared to OLIF + BPS and OLIF + UVS-PS. This result is consistent with the study by Guofang Fang et al. They found that OLIF + BPS can reduce the maximum stress on the endplate, thereby reducing the incidence of cage settlement. Compared to OLIF + BPS, the IO method of OLIF surgery generates more pressure, especially in terms of extension and flexion, which may be a potential risk factor for cage settlement (Fang et al., 2020). In addition, in our study, the maximum stress on the fusion cage during forward flexion and backward extension is significantly greater than in other directions, mostly occurring in the upper front or lower back. This indicates that the fusion cage is more likely to enter the vertebral body in an inclined posture (Figure 6). This is consistent with the research results of Fang, G. et al. (Fang et al., 2020). The maximum cage stress in the OLIF + UPS was less than that in the IO and OLIF + DRVS, but greater than that in OLIF + BPS and OLIF + UVS-PS. Under different motion loads, the cage was less stressed in the OLIF + BPS and OLIF + UVS-PS. Only in flexion and rotating right, the maximum cage stress was greater in OLIF + UVS-PS than in OLIF + BPS. In the rest of the motion loads, the maximum cage stress was similar in both internal fixation methods, indicating a lower likelihood of cage subsidence in both models.

The maximum internal fixation stresses for OLIF + UVS-PS were significantly greater in flexion, extension, and rotating left compared to the other models, except for OLIF + DRVS. These results indicate that the components of OLIF + DRVS and OLIF + UVS-PS are more likely to fracture. The maximum internal fixation stresses for OLIF + UPS and OLIF + BPS were smaller and similar in all motion directions. The maximum internal fixator stress of OLIF + BPS was greater than that of OLIF + UPS only in left rotating. Therefore, OLIF + BPS is the least likely to experience a component fracture due to metal fatigue, followed by OLIF + UPS. These findings are consistent with the study by Cai, X. Y. et al., where the maximum stress of OLIF + DRVS was significantly greater than that of OLIF + UPS and OLIF + BPS (Cai et al., 2022).

The performance of the ROM and maximum stresses of OLIF + BPS were better than the other models under the same loading conditions. This indicates that OLIF + BPS can limit the movement of the surgical segment, share the stress of the fusion and endplate, maintain the effect of indirect decompression after OLIF, and further improve its stability with significant biomechanical advantages. However, OLIF + BPS cannot be performed in a single position and requires intraoperative changes in the patient’s position. OLIF + UPS were slightly less effective than OLIF + BPS in terms of internal fixation, but still provided good biomechanical stability in all directions of motion and did not require intraoperative changes in the patient’s position. OLIF + UVS-PS is inferior to OLIF + UPS in terms of internal fixation stress, patient economic burden, and surgical procedure. Moreover, the vertebral screws of OLIF + UVS-PS were only subjected to a small amount of stress, which indicates that vertebral screws may not be necessary (Figure 8). The ROM and maximum fusion stresses of IO and OLIF + DRVS are inferior to those of the other models, which indicates that the incidence of fusion subsidence and vertebral instability may be higher in these two models than in the other models. Only one of the two vertebral screws in OLIF + DRVS was significantly stressed, and the crossbeam was almost unstressed, which indicates that it may not be necessary to add two vertebral screws to the vertebral body and connect them through the crossbeam (Figure 8).

Although the present study comprehensively investigated different implantation methods, some limitations should be acknowledged. First, only the FE analysis is performed in the present study and no cadaver study was performed, which may result in conclusions deviating from the actual situation. Second, this study did not simulate soft tissues except for the ligaments and intervertebral discs such as muscles, fascia, and fat. These soft tissues provide slight traction due to their elasticity. However, currently, there is no evidence to prove that this traction force affects the biomechanical stability after lumbar surgery. What’s more, it is conventionally believed that early postoperative lumbar spine surgery often requires limiting lumbar muscle activity. Therefore, even without simulating soft tissues like muscles, the results will not be affected (Williamson et al., 2007). Third, simplified FE models of the cage and screw are used in the present study. However, the texture on the surface of the fusion device and the thread of the screw do not affect the overall mechanical performance, so removing the texture on their surface will not affect the results of this study. In summary, further experimental research on cadaveric biomechanics is still necessary for future investigations. Despite these limitations, our research findings can still assist spinal surgeons in selecting the most suitable fixation strategy in clinical practice.

## Conclusion

It is concluded from the present study that OLIF + BPS has the best biomechanical stability, but it requires changing the patient’s position during the surgery, which reduces the simplicity of the surgery. OLIF + UPS provides adequate biomechanical stability. OLIF + UVS-PS is inferior to OLIF + UPS in terms of internal fixation stress, patient economic burden, and surgical procedure. IO and OLIF + DRVS do not have significant advantages in biomechanical stability, and may only be of value in exceptional circumstances. Based on the biomechanical analysis, OLIF + BPS is recommended for OLIF surgery. OLIF + UPS can be used as an alternative.

## Data Availability

The original contributions presented in the study are included in the article/Supplementary material, further inquiries can be directed to the corresponding author.
